# Smart Vitamin Micelles as Cancer Nanomedicines for Enhanced Intracellular Delivery of Doxorubicin

**DOI:** 10.3390/ijms222011298

**Published:** 2021-10-19

**Authors:** Na Re Ko, Sang Ju Lee, Arun Pandian Chandrasekaran, Apoorvi Tyagi, Suresh Ramakrishna, Seog-Young Kim, Do Won Kim, Chan-Gi Pack, Seung Jun Oh

**Affiliations:** 1 Biomedical Research Center, Asan Institute for Life Sciences, Asan Medical Center, 88 Olympic-ro 43-gil, Songpa-gu, Seoul 05505, Korea; nare.ko@amc.seoul.kr (N.R.K.); dowonah@naver.com (D.W.K.); 2 Department of Nuclear Medicine, Asan Medical Center, University of Ulsan College of Medicine, 88 Olympic-ro 43-gil, Songpa-gu, Seoul 05505, Korea; atlas425@amc.seoul.kr; 3 Graduate School of Biomedical Science and Engineering, Hanyang University, 222 Wangsimni-ro, Seongdong-gu, Seoul 04763, Korea; arunbio@hanyang.ac.kr (A.P.C.); apoorvityagi09@gmail.com (A.T.); suri28@hanyang.ac.kr (S.R.); 4 College of Medicine, Hanyang University, 222 Wangsimni-ro, Seongdong-gu, Seoul 04763, Korea; 5 Department of Convergence Medicine, University of Ulsan College of Medicine, 88 Olympic-ro 43-gil, Songpa-gu, Seoul 05505, Korea; sykim3@amc.seoul.kr (S.-Y.K.); changipack@amc.seoul.kr (C.-G.P.)

**Keywords:** vitamins, nanomedicines, stimuli-responsive degradation, chemotherapy, doxorubicin

## Abstract

Chemotherapy is one of the most effective treatments for cancer. However, intracellular delivery of many anticancer drugs is hindered by their hydrophobicity and low molecular weight. Here, we describe highly biocompatible and biodegradable amphiphilic vitamin conjugates comprising hydrophobic vitamin E and hydrophilic vitamin B labeled with dual pH and glutathione-responsive degradable linkages. Vitamin-based micelles (vitamicelles), formed by self-assembly in aqueous solutions, were optimized based on their stability after encapsulation of doxorubicin (DOX). The resulting vitamicelles have great potential as vehicles for anticancer drugs because they show excellent biocompatibility (>94% after 48 h of incubation) and rapid biodegradability (>90% after 2.5 h). Compared with free DOX, DOX-loaded vitamicelles showed a markedly enhanced anticancer effect as they released the drug rapidly and inhibited drug efflux out of cells efficiently. By exploiting these advantages, this study not only provides a promising strategy for circumventing existing challenges regarding the delivery of anticancer drugs but also extends the utility of current DOX-induced chemotherapy.

## 1. Introduction

Chemotherapy uses anticancer drugs to destroy or inhibit the growth of cancer cells. It is one of the most effective treatments that improve cancer survival, and it can be given before and/or after surgery, either alone or in combination with other treatments, such as hormones [1], radiation [2], and immunotherapy [3]. However, intracellular delivery of anticancer drugs is hindered by their hydrophobicity and low molecular weight. Many effective anticancer drugs have poor aqueous solubility; thus, they are recognized by opsonins and cleared by the reticuloendothelial system (RES) [4]. Small anticancer drugs enable the treatment of cancers almost anywhere in the human body. However, because they are not selective for cancer, they accumulate in normal tissues by passive diffusion, causing unpleasant side effects. One reason for the low efficiency of small hydrophobic drugs is P-glycoprotein (P-gp), a membrane transporter overexpressed by cancer cells. P-gp pumps drug molecules out of cells, a process called drug efflux [5]. Therefore, overexpression of P-gp reduces the intracellular concentration of anticancer drugs, thereby reducing the efficacy of chemotherapy [6].

To circumvent these challenges, nanomedicines have been the subject of extensive research to assess their applicability as platforms for cancer therapy [7]. Examples include polymers [8], liposomes [9], quantum dots [10], and metals [11], all of which are used to shield hydrophobic drugs from the RES; these moieties also release drugs at the target site in a controlled manner, thereby minimizing side effects and toxicity. Despite these advantages, few nanomedicines have been clinically approved [12,13]. The reasons for this are two-fold. First, most nanomedicines are not treatments for cancer; rather, they act only as vehicles for drug transportation. These limitations have led researchers to design new materials whose inherent properties make them suitable as drug carriers. For example, polymeric nanomedicines with intrinsic antioxidant properties show enhanced anticancer efficacy [14,15,16]. Second, there is a lack of knowledge about the toxicity of delivery materials. Most studies demonstrate that the biocompatibility of nanomedicine is mainly considered as the toxicity of the nanomedicine itself. However, biocompatibility is also highly dependent on the toxicity of carrier residues present after drug delivery and/or degradation. Therefore, a different approach to creating biocompatible nanomedicines is necessary to minimize the risks posed by the undesirable accumulation of degraded carrier residues in the body.

Here, we developed vitamin-based micellar drug carriers comprising vitamin E (VE) and vitamin B6 (VB) to create nanomedicines with anticancer activity. A lipid-soluble VE is an antioxidant that inhibits the production of reactive oxygen species and disrupts the mitochondrial membrane potential [17]. While the mechanism underlying mitochondria-mediated cell apoptosis via VE is unclear, recent studies report a myriad of different VE-conjugated nanomedicines as promising platforms for cancer treatment due to their potent anticancer activity and mitochondria-targeting abilities [18,19,20,21,22]. Water-soluble VB also shows strong antioxidant activity, similar to that of VE, making it attractive for use as a cancer-targeting drug delivery system [23]. First, the pyridine moiety in VB becomes protonated as the pH decreases [24]. Such a transition creates positively charged surface VB-containing drug carriers with properties that make them amenable to endosomal escape via the proton sponge effect [25,26]. In addition, cellular uptake of VB is facilitated by a specific protein called vitamin B_6_-transporting membrane carrier (VTC) [27]. Because VB is a nutrient essential for cell growth and function, fast-proliferating cancer cells require large amounts; consequently, VTC is overexpressed in cancer cells. Various VB-modified materials are internalized into cancer cells through the VTC-mediated pathway [28,29,30].

**Figure 1:**
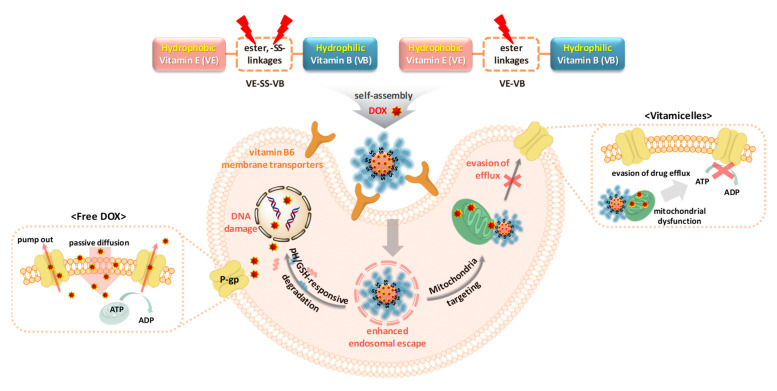
Schematic illustration of the mechanism underlying cancer cell apoptosis induced by dual stimuli-responsive degradable vitamicelles.

Based on these compelling characteristics, we selected VE and VB as the main components of our drug carrier; these were linked covalently to obtain amphiphilic conjugates (Figure 1). Such a bottom-up synthetic approach generates highly biocompatible vitamin-based nanomedicines, both before and after degradation. In an aqueous environment, the amphiphilic vitamin conjugates self-assemble to form micellar platforms (vitamicelles) comprising a hydrophobic VE core for entrapment of hydrophobic anticancer drugs and a hydrophilic VB surface for colloidal stability. In addition, stimuli-responsive degradation (SRD) is incorporated into the design of vitamin conjugates. pH-sensitive cleavable esters and/or glutathione (GSH)-responsive cleavable disulfides are positioned at the junction between VE and VB; these cleavable esters and/or glutathione (GSH)-responsive cleavable disulfides are located at the interfaces of the vitamicelles after micellization. The tumor microenvironment is more acidic than the rest of the body [31]; also, the GSH concentration in cancer cells is much higher than that in normal cells [32]. Such significant variations in pH and GSH levels are utilized to promote tumor-specific drug release by vitamicelles, thereby minimizing any toxic side effects. Furthermore, the micellar formulation of the vitamin conjugates is optimized by varying the weight ratio of the vitamin conjugates to achieve stable DOX-loaded vitamicelles. Kinetic studies of DOX release indicated that DOX-loaded vitamicelles have great potential as nanomedicines for cancer therapy. The results from in vitro experiments (cell viability assays, western blotting, confocal microscopy, fluorescence correlation spectroscopy, and ATPase activity assay) revealed that vitamicelles have excellent biocompatibility and that DOX-loaded vitamicelles plus SRD act synergistically to increase intracellular trafficking and therapeutic effects by efficiently inhibiting drug efflux.

## 2. Results and Discussion

### 2.1. Synthesis and Characterization of Stimuli-Responsive Degradable Vitamin Conjugates

**Scheme 1:**
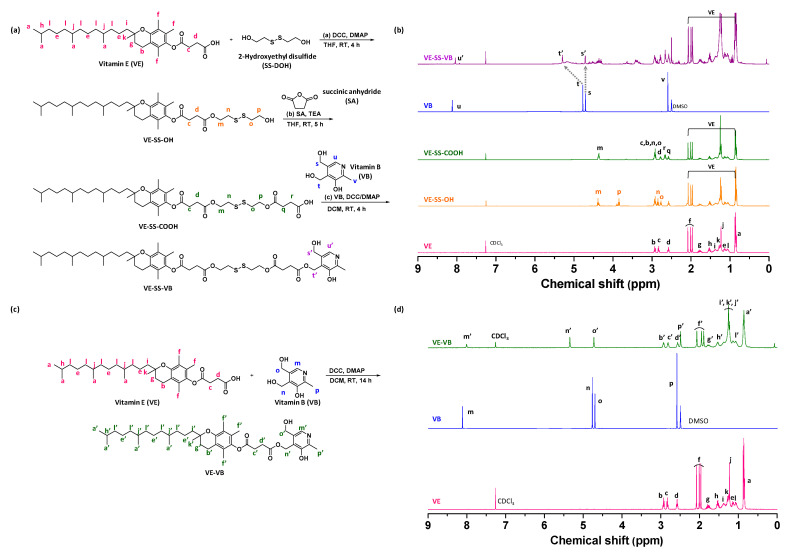
Synthesis and ^1^H-NMR spectra of VE-SS-VB (**a**,**b**) and VE-VB (**c**,**d**).

Here, we prepared two types of vitamin conjugate. First, we synthesized a dual pH and GSH-responsive degradable VE-SS-VB conjugate in three steps (Scheme 1a). A disulfide linkage was labeled with VE via a facile DCC/DMAP coupling reaction to produce VE-SS-OH. The hydroxyl group of VE-SS-OH was then carboxylated via esterification in the presence of SA. A carboxylic acid group of VE-SS-COOH acts as a coupling partner with one of the hydroxyl groups on VB. Second, we synthesized a mono pH-responsive degradable VE-VB conjugate using the DCC/DMAP click reaction between VE and VB (Scheme 1c). Successful synthesis was confirmed by ^1^H-NMR (Scheme 1b,d) and HRMS.

### 2.2. Self-Assembly and Micellar Formulation of Vitamicelles

Because the VE-SS-VB and VE-VB conjugates consist of both hydrophobic VE and hydrophilic VB, the resulting amphiphilicity enables self-assembly in an aqueous environment to generate a micellar platform. First, we examined the CMC of each VE-SS-VB and VE-VB using a fluorescent dye, NR. Briefly, each vitamin conjugate was self-assembled at various concentrations, and the fluorescence intensity of NR encapsulated in the micelle cores was monitored (Figure S1 and Table 1). Notably, incremental increases in the number of hydrophobic intra-micellar interactions increase the stability of the micelles, with a decrease in the CMC [33,34]. Compared with the VE-VB conjugate, the VE-SS-VB conjugate possesses four ester groups and one disulfide linkage at a block junction, which makes the core of the vitamicelles more hydrophilic. Consequently, the strength of the intra-micellar interaction for VE-SS-VB is weaker than that for VE-VB, which is reflected in the increased CMC value. To optimize the stability of the vitamicelles, the CMCs of the vitamin conjugate mixtures at different weight ratios (VE-SS-VB:VE-VB = 1:1 or 1:2) were explored as described above. Interestingly, the CMC value decreased in the presence of VE-VB. When the VE-SS-VB and VE-VB conjugates were mixed at a 1:1 wt/wt ratio (VT-SS-MC), the CMC was 138.9 μg/mL, with good stability, whereas at a 1:2 wt/wt ratio, the CMC was 170.3 μg/mL, with low stability. These fundamental studies suggest that VT-SS-MC vitamicelles are a prospective candidate for drug delivery.

**Figure 2:**
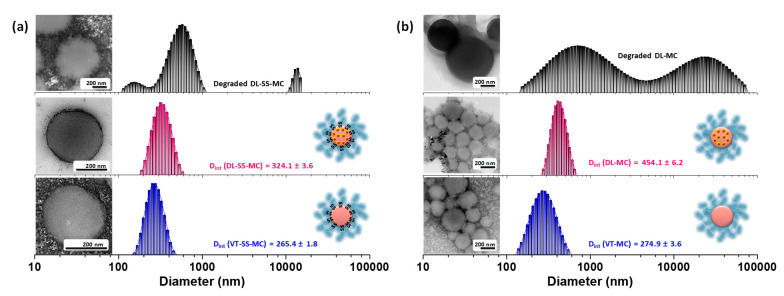
(**a**) DLS diagrams and TEM images of VT-SS-MC, DL-SS-MC, and degraded DL-SS-MC at pH 5.5/37 °C in the presence of 10 mM GSH. (**b**) DLS diagrams and TEM images of VT-MC, DL-MC, and degraded DL-MC at pH 5.5/37 °C. Scale bar = 200 nm.

At concentrations above the CMC, colloidally stable VT-SS-MC and VT-MC amphiphilic vitamicelles were formed. The size and morphology of the empty vitamicelles, as determined by DLS and TEM, are depicted in Figure 2a (VT-SS-MC) and Figure 2b (VT-MC). The DLS results indicate that VT-SS-MC and VT-MC have a similar hydrodynamic diameter (265.4 ± 1.8 nm vs. 274.9 ± 3.6 nm, respectively), with a monomodal size distribution. The TEM images indicate spherical vitamicelles with an average diameter of 210.8 ± 4.1 nm (VT-SS-MC) and 213.3 ± 5.7 nm (VT-MC). Note that the size of the micelles measured by TEM is smaller than that measured by DLS; this is because TEM measurements are carried out using dehydrated micelles [35]. In addition, similar sizes of VT-SS-MC and VT-MC were observed after storage for two months, suggesting their long-term storage stability (Table S1).

### 2.3. Biocompatibility of Empty Vitamicelles

**Figure 3:**
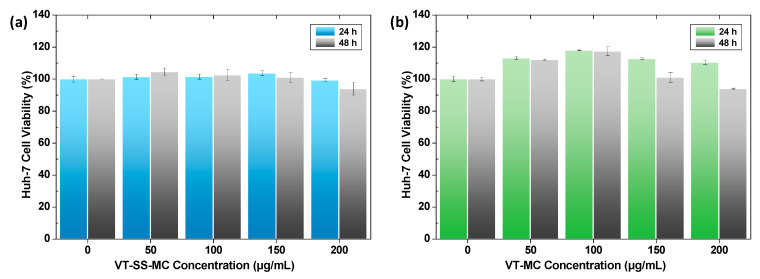
The viability of Huh-7 cells incubated with empty VT-SS-MC (**a**) and VT-MC vitamicelles (**b**) for 24 or 48 h was determined by CellTiter-Glo^®^ luminescent cell viability assay. Data are presented as the mean ± SD of triplicate experiments.

Biocompatibility is a critical property of nanomedicines used for clinical applications. First, we examined the in vitro cytotoxicity of Huh-7 cells incubated with VT-SS-MC or VT-MC (0, 50, 100, 150, or 200 μg/mL; Figure 3). The viability of Huh-7 cells remained high (>94% after 48 h) after incubation with either of the vitamicelles at concentrations up to 200 μg/mL. Furthermore, the response of Huh-7 and MCF-7 cells to 0, 25, 50, and 100 nM of the empty micelles were visualized by staining with ethidium homodimer-1 (EthD-1) followed by analyses of the dead cell population (Figure S2). No significant toxicity was observed in the presence of either vitamicelle at concentrations up to 100 nM. These results demonstrate that VT-SS-MC and VT-SS-MC show excellent biocompatibility, indicating that they are physiologically acceptable as drug delivery carriers.

### 2.4. Loading and Stimuli-Responsive Release of DOX

Next, to examine the potential of VT-SS-MC vitamicelles as effective nanomedicines for cancer therapy, we prepared doxorubicin (DOX)-loaded VT-SS-MC vitamicelles (DL-SS-MC) and monitored their pH/GSH-triggered release. DOX, a poorly water-soluble UV-sensitive anticancer drug, was encapsulated within the hydrophobic core of DL-SS-MC. The DLS and TEM results indicate that the diameter of the DL-SS-MC (324.1 ± 3.6 nm) was greater than that of the empty VT-SS-MC (265.4 ± 1.8 nm), with no significant change in spherical morphology (Figure 2a). The same trend was observed with DOX-loaded VT-MC vitamicelles (DL-MC) and empty VT-MC (Figure 2b). The increase in the diameter size of DOX-loaded vitamicelles is attributed to expansion driven by successful encapsulation of DOX within the hydrophobic core [36]. Additionally, DL-SS-MC and DL-MC exhibit long-term stability for two months (Table S1). The LC and EE of DOX within DL-SS-MC and DL-MC were investigated by UV-Vis spectroscopy. First, the extinction coefficient (ε) of DOX was determined from a calibration curve of absorbance at λ_max_ = 498 nm versus the concentration of DOX in a mixture of DW/DMF (1/3 (*v*/*v*); Figure S3). Using the Beer-Lambert equation, the LC and EE of DL-SS-MC were calculated as 6.7 ± 0.7% and 57.1 ± 12.9%, respectively. Following the same procedure, the LC and EE of DL-VT were calculated as 6.3 ± 0.8% and 50.5 ± 12.2%, respectively.

**Figure 4:**
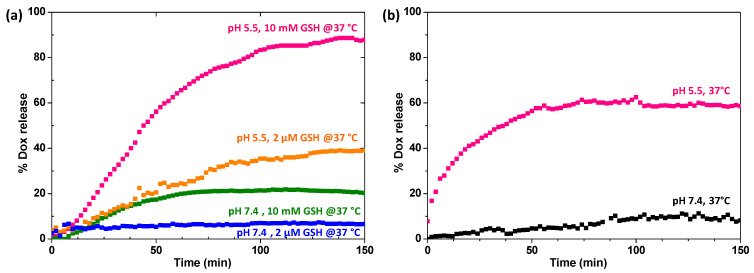
Release profile of DOX from DOX-loaded vitamicelles under different conditions. (**a**) DOX release from DL-SS-MC in PBS buffer at 37 °C at pH 5.5/10 mM GSH; pH 5.5/2 μM GSH; pH 7.4/10 mM GSH; and pH 7.4/2 μM GSH. (**b**) DOX release from DL-MC in PBS buffer at 37 °C at pH 5.5/pH 7.4.

The resulting DL-SS-MCs were examined for their ability to release the encapsulated DOX in response to low pH and high levels of GSH. To mimic the tumor and physiological environments, DL-SS-MC was exposed to the following conditions at 37 °C: pH 5.5 and 10 mM GSH; pH 5.5 and 2 μM GSH; pH 7.4 and 10 mM GSH; and pH 7.4 and 2 μM GSH. As shown in Figure 4a, >90% of the DOX was released rapidly (within 150 min) from the DL-SS-MC at pH 5.5 in the presence of 10 mM GSH, without any evidence of early burst release. Note that such fast drug release is a unique advantage of small molecule-based carriers [37,38,39] In particular, >40% of the DOX was released at pH 5.5 in the presence of 2 μM GSH, which is almost twice that released at 10 mM GSH/pH 7.4 (~20%). This implies that DL-SS-MC is more affected by pH than by the GSH concentration. Note that DL-SS-MC comprises both dual SRD (pH and GSH) VE-SS-VB and mono SRD (pH) VE-VB conjugates. These structural characteristics mean that DL-SS-MC is more sensitive to pH than it is to the GSH concentration. In the presence of 2 μM GSH/pH 7.4, little DOX (<10%) was released, confirming that DL-SS-MC would be stable in circulating blood under physiological conditions. For comparison, we examined the DOX release profile of the mono pH-responsive degradable DL-VT under different pH conditions (5.5 and 7.4) at 37 °C. As seen in Figure 4b, about 60% of the DOX was released from DL-MC within 150 min at pH 5.5, while only 10% was released at pH 7.4. These results demonstrate that DL-SS-MC is a promising platform because it prevents premature release of DOX at undesired sites while enabling controlled and rapid release at the target sites. Such on-demand degradation of DL-SS-MC can minimize the possible toxic effects of FD in vivo.

Upon degradation, in response to a tumor-mimicking environment, the surface roughness of the DOX-loaded vitamicelles increases with the formation of large aggregates (Figure 2a,b). These changes in size and morphology suggest that DOX release is due to cleavage of the ester/disulfide linkages at the interfaces of the vitamicelles, leading to complete dissociation of DL-SS-MC and DL-MC.

### 2.5. Effective Anticancer Activity of DL-SS-MC

**Figure 5:**
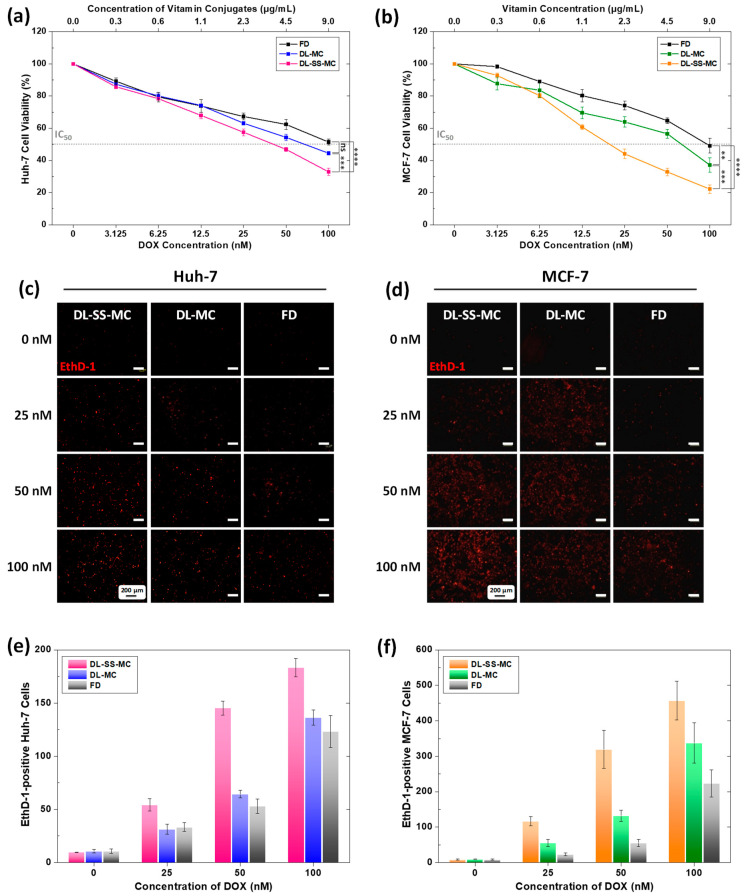
Viability of Huh-7 (**a**) and MCF-7 cells (**b**) treated with DL-SS-MC, DL-MC, and FD for 24 h, as measured in a CCK-8 assay. Data are presented as the mean ± SD of triplicate experiments: ns (not statistically significant, *p* > 0.05), ** *p* < 0.01, *** *p* < 0.001, **** *p* < 0.0001. Fluorescence microscopy images of EthD-1-stained Huh-7 cells (**c**) and MCF-7 cells (**d**) incubated with DL-SS-MC, DL-MC, and FD for 24 h (scale bar = 200 μm). The number of dead Huh-7 cells (**e**) and MCF-7 cells (**f**) were quantified.

To investigate the anticancer activity of DL-SS-MC and DL-MC in vitro, we incubated them with Huh-7 and MCF-7cells, which are representative cancer cell lines used to test DOX-mediated chemotherapy. First, we evaluated in vitro cytotoxicity by comparing the viability of cells exposed to DL-SS-MC, DL-MC, or FD. The biocompatibility experiments showed that the toxicity of the vitamin conjugates themselves is negligible; therefore, the amount of DOX-loaded vitamicelles used for treatment was based on the concentration of DOX, and the amount of FD was adjusted such that it was equivalent to the amount of DOX encapsulated in the DOX-loaded vitamicelles. As shown in Figure 5a, the viability of Huh-7 cells fell significantly, to 32%, in the presence of 100 nM DOX encapsulated in DL-SS-MC, while viability was 51% in the presence of 100 nM FD. Similarly, the viability of MCF-7 cells fell sharply, to 22%, in the presence of 100 nM DOX encapsulated in DL-SS-MC, while 49% viability was observed in the presence of 100 nM FD (Figure 5b). Specifically, MCF-7 cells were more sensitive to DL-SS-MC than Huh-7 cells. Compared with that in MCF-7 cells, the IC_50_ of DL-SS-MC in Huh-7 cells was almost 2-fold higher, whereas that of FD in both cell lines was similar. In addition, the viability of both Huh-7 and MCF-7 cells in the presence of DL-MC was higher than that in the presence of DL-SS-MC. This is due to the greater release of DOX from the dual pH and GSH-triggered degradable DL-SS-MC than from the mono pH-triggered degradable DL-MC. Figure 5c,d show Huh-7 and MCF-7 cells incubated with DL-SS-MC, DL-MC, and FD for 24 h and stained with EthD-1. The population of dead cells showed a positive association with cytotoxicity in both cancer cell lines (Figure 5e,f). Taken together, these results demonstrate that DL-SS-MC has great potential to circumvent the limitations of FD by effectively inhibiting cancer cell proliferation.

### 2.6. Increased DOX-Induced DNA Damage and Apoptosis

**Figure 6:**
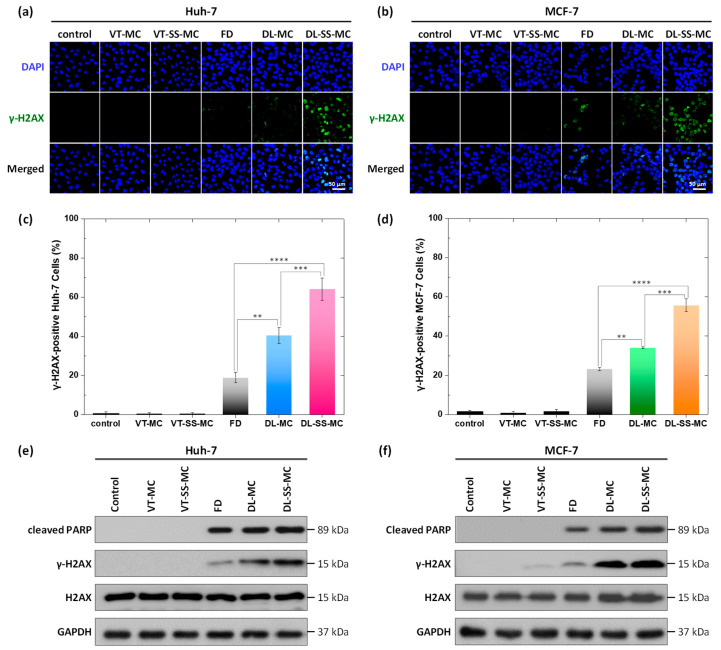
Huh-7 and MCF-7 cells were treated with empty vitamicelles (VT-MC, VT-SS-MC), DOX-loaded vitamicelles (DL-MC, DL-SS-MC), or FD for 24 h. (**a**,**b**) Fluorescence microscopy images of both cancer cell lines. Blue and green fluorescence represent cell nuclei (DAPI) and a marker of DNA double-strand breaks (γ-H2AX), respectively. Scale bar = 50 μm. (**c**,**d**) Number of γ-H2AX-positive cancer cells. Data are presented as the mean ± SD: ** *p* < 0.01, *** *p* < 0.001, **** *p* < 0.0001. (**e**,**f**) Western blot analysis of PARP, γ-H2AX, and H2AX expression in both cancer cell lines after treatment. GAPDH served as a loading control.

DOX inhibits topoisomerase II by interfering with the topoisomerase II-DNA complex [40]. This primary mechanism of DOX activity involves DNA double-strand breaks or direct intercalation into DNA, leading to apoptosis [41]. First, we examined the effects of DOX-loaded vitamicelles on DOX-induced DNA damage in cancer cells using a marker for DNA double-strand breaks, phosphorylated H2AX (γ-H2AX). When DNA is damaged, H2AX is phosphorylated within minutes and accumulates to form nuclear foci [42]. No DNA damage was observed when cells were incubated with empty VT-SS-MC and VT-MC vitamicelles (Figure S4). As shown in Figure 6a–d, the expression of γ-H2AX increased significantly in both Huh-7 and MCF-7 cells treated with DOX-loaded vitamicelles compared with that in cells treated with FD. More importantly, a higher expression of γ-H2AX was triggered by DL-SS-MC than by DL-MC. These results suggest that the fast release of DOX from the dual stimuli-responsive DL-SS-MC effectively triggers DNA damage.

Apoptosis is a prominent route of cell death caused by DNA damage [43]. To examine the extent of DNA damage-mediated apoptosis induced by DOX-loaded vitamicelles, we conducted western blot analysis to examine the expression of γ-H2AX, H2AX, and cleaved PARP (Figure 6e,f). In both Huh-7 and MCF-7 cancer cell lines, the expression of γ-H2AX increased markedly when they were treated with DOX-loaded vitamicelles compared with FD. In addition, cleaved PARP was upregulated to a greater extent following treatment with DL-SS-MC than after treatment with DL-MC. All of these findings are consistent with the results from the cytotoxicity and DNA damage experiments, indicating that DL-SS-MC is a promising nanomedicine with the low cytotoxicity associated with DNA damage-induced apoptosis.

### 2.7. Enhanced Intracellular Trafficking and Colocalization of DOX

**Figure 7:**
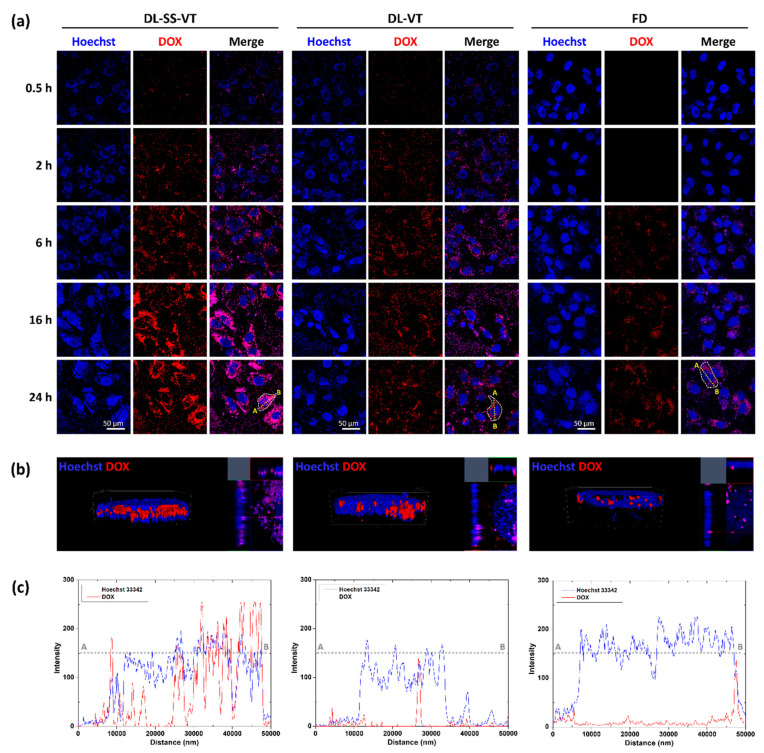
(**a**) CLSM images of Huh-7 cells incubated with DL-SS-MC, DL-MC, or FD for 0.5, 2, 6, 16, or 24 h. Blue and red fluorescence represent cell nuclei (Hoechst 33342) and DOX, respectively. Scale bar = 50 μm. (**b**) 3D reconstructions and Z-stack images (left and right side of each set, respectively) of Huh-7 cells incubated with DL-SS-MC, DL-MC, or FD for 24 h. (**c**) Localization of DOX in the cell nuclei is confirmed by intensity overlay of the fluorescence signals.

The results described above suggest that apoptosis of DL-SS-MC is mediated by DOX. However, delivery of DOX by DL-SS-MC promotes apoptosis more strongly than delivery of free DOX (i.e., without a carrier). We hypothesize that increased apoptosis is related to increased intracellular trafficking of DL-SS-MC. To validate this, we used CLSM to visualize the internalization and accumulation of DOX in cancer cells. DL-SS-MC, DL-MC, and free DOX were incubated with Huh-7 cells, and uptake was observed at different time points (0.5, 2, 4, 16, and 24 h). CLSM images demonstrate that the DOX signal in all samples increased gradually over time, reaching maximum intensity after 16 h (Figure 7a). Promisingly, DOX intensity was higher after treatment with DL-SS-MC than after treatment with DL-MC, indicating that the dual pH and GSH-responsiveness increase the degradation of the vitamicelles to allow rapid DOX release. In addition, DOX intensity was stronger after treatment with DL-SS-MC and DL-MC than after treatment with free DOX. The intracellular distribution of DOX after 24 h was readily visualized by reconstructing 3D images (Figure 7b) and by intensity overlays of the fluorescence signals (Figure 7c). These results indicate that DOX released from DL-SS-MC and DL-MC localized mainly in the cell nucleus; the high intranuclear accumulation of DOX released from DL-SS-MC increases cancer cell apoptosis.

### 2.8. Inhibition of Drug Efflux by Decreasing ATPase Activity

**Figure 8:**
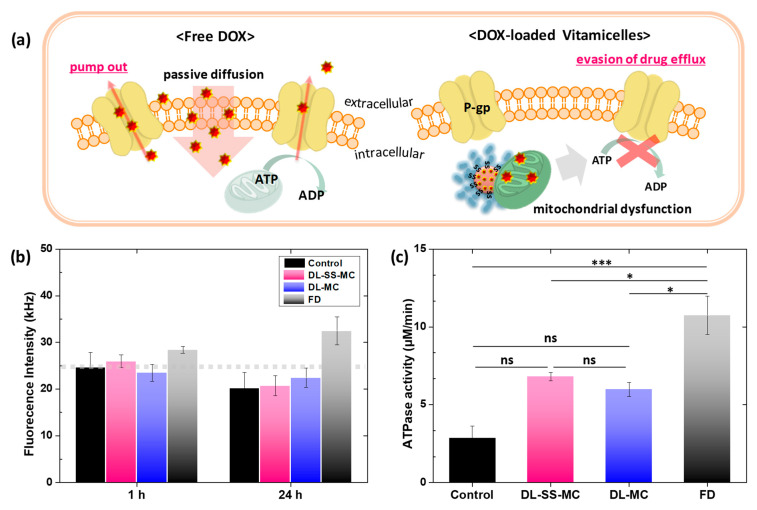
(**a**) Schematic illustration of the drug efflux mechanism that removes free DOX (**a**), and intracellular trafficking of DOX-loaded vitamicelles in cancer cells. (**b**) Fluorescence intensity of DOX in the extracellular fluid around Huh-7 cells after incubation with DL-SS-MC, DL-MC, or FD for 1 or 24 h. (**c**) ATPase assay to test drug efflux from Huh-7 cells incubated with DL-SS-MC, DL-MC, or FD. Data are presented as the mean ± SD of triplicate experiments: ns (not statistically significant, *p* > 0.05), * *p* > 0.05, *** *p* < 0.001.

Small DOX molecules are readily pumped out of cells, thereby decreasing the intracellular concentration of DOX; this mechanism is reflected by the low fluorescence intensity of free DOX. Based on the CLSM results presented above, we hypothesized that vitamicelles inhibit P-gp-mediated drug efflux (Figure 8a). To evaluate the effect of DOX-loaded vitamicelles on drug efflux, we incubated Huh-7 cells with DL-SS-MC, DL-MC, or free DOX for 24 h and monitored the fluorescence intensity of DOX in the extracellular fluid by fluorescence correlation spectroscopy. As shown in Figure 8b, the fluorescence intensity of free DOX was higher than that of DOX-loaded vitamicelles after 1 h of incubation. This difference was more obvious after 24 h. While there was a decrease in the intensity of DOX-loaded vitamicelles, there was a significant increase in the intensity of free DOX. These results suggest that the vitamicelles successfully inhibit DOX efflux, thereby increasing the DOX concentration in cancer cells.

Drug efflux is a dynamic mechanism that requires energy consumption via hydrolysis of adenosine triphosphate (ATP) by ATPase. Because ATP is produced by mitochondrial oxidative phosphorylation, and because a lack of ATP provokes dysfunction of P-gp pumps, we hypothesized that ATP levels are modulated by inhibition of drug efflux by vitamicelles containing the mitochondria-targeting VE component. To test this, we conducted an ATPase assay of drug efflux across the membrane in the presence of DL-SS-MC, DL-MC, or FD (Figure 8c). After 24 h, Huh-7 cells incubated with DL-SS-MC and DL-MC showed significantly lower levels of ATPase activity than cells incubated with FD. This result is comparable to flow cytometry results showing that vitamicelles inhibit drug efflux. Taken together, the results suggest that vitamicelles reduce ATP production, thereby suppressing P-gp-mediated drug efflux and increasing the intracellular concentration of DOX.

## 3. Experimental Section

### 3.1. Materials

D-α-Tocopherol succinate (vitamin E succinate, VE, ≥ 98%), pyridoxine hydrochloride (vitamin B6, VB, ≥98%), 2-hydroxyethyl disulfide (SS-DOH, <90%), succinic anhydride (SA, ≥99%), triethylamine (Et_3_N, ≥99%), N,N′-dicyclohexylcarbodiimide (DCC, 99%), 4-(N,N-dimethylamino)pyridine (DMAP, >98%), glutathione (GSH, reduced form, ≥98%), Nile Red (NR), and doxorubicin hydrochloride (DOX, -NH^3+^Cl^−^ forms, >98%) were purchased from Sigma-Aldrich (St. Louis, MO, USA). Roswell Park Memorial Institute (RPMI) 1640, Dulbecco’s modified Eagle’s medium (DMEM), fetal bovine serum (FBS), and Trypsin-EDTA (TE) were purchased from Hyclone (Logan, UT, USA). Dulbecco’s phosphate-buffered saline and penicillin-streptomycin (PS) were purchased from GIBCO BRL (Grand Island, NY, USA). Unless indicated otherwise, chemicals were purchased and used without further purification.

### 3.2. Instrumentation

^1^H-NMR spectra were recorded using a Bruker Avance HD 400 (400 MHz). The CDCl_3_ singlet at 7.26 ppm and the DMSO-*d*_6_ multiplet at 2.5 ppm were selected as reference standards. High-resolution mass spectrometry (HRMS) was recorded on a Synapt G2 (Waters MS Technology, Manchester, UK). UV-Visible spectra were monitored using an Agilent Cary 60 UV-Vis spectrometer, and fluorescence spectra were measured using an Infinite M200pro microplate reader (Tecan; Männedorf, Switzerland). The hydrodynamic diameter of vitamicelles was measured by dynamic light scattering (DLS; ELSZ-1000; Otsuka Electronics, Osaka, Japan), and the morphology and core size of the vitamicelles were observed by field-emission transmission electron microscopy (FE-TEM; JEOL 2100F; Tokyo, Japan). To prepare specimens for FE-TEM, aqueous vitamicelle dispersions were dropped onto copper TEM grids (400 mesh, carbon-coated), blotted, and allowed to air dry at room temperature. The diameter of the vitamicelles was measured using ImageJ software (version 1.53c; National Institutes of Health; Bethesda, MD, USA).

### 3.3. Synthesis of VE-SS-OH

SS-DOH (1.60 g, 10.4 mmol), dissolved in tetrahydrofuran (THF, 10 mL), was added dropwise at 0 °C to a solution comprising VE (5 g, 9.42 mmol), DMAP (138 mg, 1.13 mmol), and THF (30 mL) under stirring. DCC (2.33 g, 11.3 mmol), dissolved in THF (10 mL), was added to the mixture and stirred at room temperature for 4 h. White solids (dicyclohexylurea, DCU) formed during the reaction were filtered through Celite. The filtrate was concentrated by rotary evaporation and diluted in ethyl acetate (EA). The residue was washed three times with distilled water, and the organic layer was dried with anhydrous Na_2_SO_4_. EA was then removed by rotary evaporation, and the product was purified by silica column chromatography with EA/hexane (HX; 3/7 (*v*/*v*)). The yield was 3.16 g (50.2%). HRMS-ESI (*m*/*z*): [M + Na]^+^ Calcd. for C_37_H_62_O_6_S_2_Na: 689.3885; Found at 689.3909.

### 3.4. Synthesis of VE-SS-COOH

A solution comprising purified VE-SS-OH (3.16 g, 4.73 mmol) and Et_3_N (0.957 g, 8.53 mmol) in THF (40 mL) was mixed with a solution of SA (0.947 g, 9.46 mmol) in THF (10 mL) at 0 °C, and then stirred at room temperature for 5 h. The reaction mixture was filtered to remove the white solids, and the resulting VE-SS-COOH was obtained as a crude product. HRMS-ESI (*m*/*z*): [M + Na]^+^ Calcd. for C_41_H_66_O_9_S_2_Na: 789.4046, found at 789.4080.

### 3.5. Synthesis of VE-SS-VB Conjugates

VE-SS-COOH (3.60 g, 10.4 mmol), dissolved in DCM (40 mL), was added dropwise at 0 °C to a solution comprising VB (1.45 g, 7.04 mmol), DMAP (1.13 g, 9.22 mmol), and DCM (50 mL) under stirring. DCC (1.60 g, 7.74 mmol), dissolved in DCM (30 mL), was added, and the mixture was stirred at room temperature for 4 h. DCU was filtered through Celite, and the filtrate was concentrated by rotary evaporation. The residue was diluted in EA and washed three times with distilled water. The organic layer was dried with anhydrous Na_2_SO_4_. The product was purified by silica column chromatography with a mixture of DCM/EA/MeOH (45/50/5 (*v*/*v*)). The yield was 1.61 g (37.3%). HRMS-ESI (*m*/*z*): [M + H]^+^ Calcd. for C_49_H_76_NO_11_S_2_: 918.4860, found at 918.4875.

### 3.6. Synthesis of VE-VB Conjugates

VE (5.00 g, 9.42 mmol) was dissolved in DCM (40 mL) and then added drop-wise under stirring at 0 °C to a solution comprising VB (2.91 g, 14.1 mmol), DMAP (2.73 g, 22.3 mmol), and DCM (100 mL). DCC (1.60 g, 7.74 mmol) was dissolved in DCM (30 mL) and then added to the solution, with continued stirring at room temperature for 14 h. The DCU was filtered through Celite, and the filtrate was concentrated by rotary evaporation. The residue was then diluted in EA and washed three times with distilled water. The organic layer was dried with anhydrous Na_2_SO_4_. The product was purified by silica column chromatography using a mixture of DCM/EA/MeOH (45/50/5 (*v*/*v*)). The yield was 1.48 g (23.0%). HRMS-ESI (*m*/*z*): [M + H]^+^ Calcd. for C_41_H_64_NO_7_: 682.4683, Found at 682.4695.

### 3.7. Preparation of Empty Vitamicelles Using the Solvent Evaporation Method

A stock solution of vitamin conjugates mixed at different weight ratios (VE-SS-VB:VE-VB = 0:1, 1:2, 2:1, 1:0) was prepared in THF. Distilled water (10 mL) was added dropwise to a solution comprising vitamin conjugates (10 mg) dissolved in THF (1 mL). The resulting dispersion was stirred at room temperature to remove THF and then filtered through a 0.45 μm PES filter. The yield of the colloidal dispersion of empty vitamicelles was 0.58 ± 0.02 mg/mL.

### 3.8. Preparation of DOX-Loaded Vitamicelles Using the Dialysis Method

A stock solution of vitamin conjugates (VE-SS-VB:VE-VB = 0:1 (1:1 (wt/wt)) dissolved in THF (5 mg/mL) was added to a mixture of DOX (0.5 mg) and Et_3_N (0.36 μL, 3 molar equivalents with respect to DOX) in DMF (1 mL). Distilled water (10 mL) was added to the solution in a dropwise manner, followed by stirring at room temperature for 5 h. Then, the mixture was stirred under a fume hood for 15 h to remove THF. The pre-formed DOX-loaded micelles were transferred into a dialysis membrane (MWCO = 1000 g/mol) and dialyzed against distilled water for 20 h. The final DOX-loaded vitamicelles, comprising a mixture of VE-SS-VB:VE-VB at 0:1 and 1:1 (wt/wt), were named DL-MC and DL-SS-MC, respectively.

UV-Vis spectroscopy was used to determine the loading capacity (LC) and encapsulation efficiency (EE) of DOX in the DOX-loaded vitamicelles. To do this, a calibration curve of absorbance (λ_max_ = 498 nm) versus various concentrations of DOX in a mixture of water/DMF (1/3 (*v*/*v*)) was constructed as follows. A stock solution of DOX in DMF (0.5 mg/mL) was prepared by dissolving DOX (2 mg) in DMF (10 mL). Aliquots of the stock solution were diluted with DMF to form a series of solutions containing different concentrations of DOX (0 to 70 μM). The total volume of the DMF solutions was adjusted to 3 mL, and water (1 mL) was added to each solution. The UV-Vis spectra for each sample were recorded from 200–800 nm. Next, the LC and EE of DOX were determined as follows: first, aliquots of aqueous DOX-loaded vitamicelles were mixed with DMF (1:3 *v*/*v*). Next, the amount of encapsulated DOX was calculated using the Beer-Lambert equation at an absorbance λ_max_ = 498 nm and an extinction coefficient ε = 10,680 M^−1^ cm^−1^. The LC and EE of DOX-loaded vitamicelles were calculated as follows:

$$\mathrm{LC}=\frac{\mathrm{weight}\;\mathrm{of}\;\mathrm{DOX}\;\mathrm{in}\;\mathrm{DOX}-\mathrm{loaded}\;\mathrm{vitamicelles}}{\mathrm{total}\;\mathrm{weight}\;\mathrm{of}\;\mathrm{DOX}-\mathrm{loaded}\;\mathrm{vitamicelles}}$$

$$\mathrm{EE}=\frac{\mathrm{weight}\;\mathrm{of}\;\mathrm{DOX}\;\mathrm{in}\;\mathrm{DL}-\mathrm{vitamicelles}}{\mathrm{initial}\;\mathrm{amount}\;\mathrm{of}\;\mathrm{DOX}\;\mathrm{added}}$$

### 3.9. DOX Release from DOX-Loaded Vitamicelles

An aliquot of DL-SS-MC (3 mL) was placed into dialysis tubing (MWCO = 1000 g/mol) and dialyzed against 50 mL PBS at 37 °C under the following conditions: 10 mM GSH at pH 5.5; 10 mM GSH at pH 7.4; 2 μM GSH at pH 5.5; and 2 μM GSH at pH 7.4. For comparison, an aliquot of DL-MC (3 mL) was placed into dialysis tubing (MWCO = 1000 g/mol) and dialyzed against 50 mL PBS buffer at pH 5.5 or pH 7.4 at 37 °C. The absorbance of DOX in the outer water was recorded at 2 min intervals using a UV-Vis spectrometer equipped with an external probe (λ = 498 nm). All experiments were conducted in the dark to prevent the decomposition of DOX.

### 3.10. Cell Culture

Michigan cancer foundation 7 (MCF-7, human breast cancer) and human hepatocellular carcinoma (Huh-7) cell lines were purchased from the American Type Culture Collection (ATCC, Manassas, VA, USA). MCF-7 and Huh-7 cells were cultured in a DMEM growth medium supplemented with 10% FBS and 1% PS at 37 °C in a humidified atmosphere with 5% CO_2_. The cells were passaged every 2–3 days depending on the degree of confluence.

### 3.11. Cell Viability

The effect of empty vitamicelles on cell viability was measured using the CellTiter-Glo^®^ luminescent cell viability assay. Briefly, Huh-7 cells were plated at a density of 2 × 10^4^ cells per well into a 96-well plate and incubated for 24 h in DMEM (90 µL) containing 10% FBS and 1% antibiotics. After incubation, VT-SS-MC and VT-MC at 0, 50, 100, 150, and 200 μg/mL were added and incubated at 37 °C for 24 or 48 h. Next, CellTiter-Glo^®^ reagent (Promega) was added, and luminescence was measured using a VICTOR3 multilabel plate reader (PerkinElmer). Data were calculated and plotted using PRISM software. The effect of DOX-loaded vitamicelles on cell viability was measured in a CCK-8 assay. Briefly, Huh-7 and MCF-7 cells were plated at a density of 5 × 10^3^ cells per well into a 96-well plate and incubated for 24 h in DMEM (100 µL) containing 10% FBS and 1% antibiotics. After incubation, a solution of DL-SS-MC, DL-MC, and free DOX (FD; at different concentrations) was added for 24 h. Next, 100 µL CCK-8 solution (product #CK04; Dojindo Molecular Technologies) was added to each well, and the plate was incubated for 1 h. Absorbance was measured at 450 nm using a Benchmark Plus microplate spectrophotometer (BR170-6930; Bio-Rad; Hercules, CA, USA), and values were recorded graphically.

### 3.12. Dead Cell Staining Using Ethidium Homodimer-1

The total population of dead cells was determined by ethidium homodimer-1 (EthD-1) staining (product #L3224; Thermo Fisher; Waltham, MA, USA). Briefly, Huh-7 and MCF-7 cells were treated with 0, 25, 50, and 100 nM of FD, DOX-loaded vitamicelles (DL-MC or DL-SS-MC). At 24 h post-treatment, 150 µL PBS containing 4 µM EthD-1 was added to the treated cells and incubated at room temperature for 30 min. All images were captured under an inverted fluorescence microscope (IX71; Olympus, Tokyo, Japan).

### 3.13. γ-H2AX Foci Formation Assay

Huh-7 and MCF-7 cells were seeded onto glass coverslips and placed in a 4-well culture dish. After 24 h, cells were treated with FD, DL-MC, DL-SS-MC, VT-MC, and VT-SS-MC. Cells were then fixed for 15 min with 4% paraformaldehyde (product #30525-89-4; Wako, Osaka, Japan)), followed by washing three times (each for 5 min) in 0.03% Triton-X 100 (product #T8787; Sigma-Aldrich; St. Louis, MO, USA). Cells were blocked for 1 h using 5% BSA (product #BSAS-AU; Bovogen Biologicals; Melbourne, Australia) in PBS prior to the addition of an γ-H2AX-specific antibody (1:200; product #05-636; Millipore; Billerica, MA, USA). After incubation overnight at 4 °C, cells were washed three times with PBS prior to the addition of fluorescence-conjugated secondary antibodies (1:250; product #A20181; Invitrogen; Carlsbad, CA, USA) for 1 h at room temperature. Cells were then stained for 15 min with DAPI (product #D1306; Invitrogen; Carlsbad, CA, USA) and mounted on glass slides using an antifade mounting medium (product #H-1000-10; Vector Laboratories; Burlingame, CA, USA). Cells were visualized under a confocal microscope (Leica TCS SP5; Wetzlar, Germany).

### 3.14. Western Blot Analysis

Western blot analysis of Huh-7 and MCF-7 cells was performed to check the expression of proteins associated with DNA damage and apoptosis. Briefly, cells were seeded at a density of 3 × 10^5^ cells per well in a 6-well dish and incubated overnight. Cells were then treated with VT-MC, VT-SS-MC, DL-MC, DL-SS-MC, or FD for 24 h and subjected to western blot analysis. Diluted dispersions of DL-SS-MC and DL-MC, based on the equivalent amount of 100 nM DOX, were prepared. The calculated concentration of empty micelles was based on the equivalent amount of vitamin conjugates in the DOX-loaded vitamicelles. After 24 h, cells were harvested and lysed with protein lysis buffer (20 mM Tris-HCl pH 7.5, 150 mM NaCl, 1 mM Na_2_EDTA, 1 mM EGTA, 1% NP-40, 1% sodium deoxycholate, 2.5 mM sodium pyrophosphate, 1 mM b-glycerophosphate, 1 mM Na_3_VO_4_, and 1 µg/mL leupeptin) containing PMSF protease inhibitor (product #8553; Cell Signaling Technology; Beverly, MA, USA). The protein concentration was determined in a Bradford assay (product #5000006; Bio-Rad; Hercules, CA, USA). Then, 50 µg protein was loaded onto 8% or 12% SDS-PAGE gels and transferred to PVDF membranes (product #IPVH00010; Millipore; Billerica, MA, USA) activated by methanol. Subsequently, non-specific binding was blocked by incubation in 5% skim milk for 1 h at room temperature. The membranes were probed with the following primary antibodies (diluted 1:1000) and incubated overnight at 4 °C. The following primary antibodies were used: γ-H2AX (product #05-636; Millipore; Billerica, MA, USA), cleaved PARP (product #5625S; Cell Signaling Technology; Beverly, MA, USA), H2AX (product #sc-517336; Santa Cruz Biotechnology; Santa Cruz, CA, USA), and GAPDH (product #sc-32233; Santa Cruz Biotechnology; Santa Cruz, CA, USA). Subsequently, the membranes were incubated for 1 h at room temperature with an HRP-conjugated secondary antibody and then washed three times with TBS-Tween 20 (0.05%; each for 5 min) to remove unbound probes. The following secondary antibodies were used: goat anti-mouse IgG secondary antibody (product #31430; Invitrogen; Carlsbad, CA, USA) and goat anti-rabbit IgG secondary antibody (product #31460; Invitrogen; Carlsbad, CA, USA). The membranes were then subjected to chemiluminescence-based detection (product #32134; Thermo Fisher; Cleveland, OH, USA). Band intensity was analyzed using a ChemiDoc system and Image Lab software version 5.2 (Bio-Rad; Hercules, CA, USA).

### 3.15. Confocal Laser Scanning Microscopy (CLSM)

A total of 1 × 10^4^ Huh-7 cells per well were seeded into a 96-well optical-bottom plate (Thermo Scientific; Cleveland, OH, USA) and incubated at 37 °C for 24 h. They were then treated with DL-SS-VT, DL-VT, FD (equivalent DOX dose, 4 μM), or PBS (control) at 37 °C at different time points (0.5, 2, 6, 16, and 24 h). For fluorescence microscopy, nucleic acids were counterstained by adding 1–2 drops per well of Hoechst 33342 (Thermo Fisher Scientific; Waltham, MA, USA). The fluorescence intensity of live cells was observed under an inverted CLSM (LSM880 with Airyscan; Carl Zeiss; Oberkochen, Germany) as previously reported [44] To obtain 3D images, Huh-7 cells were illuminated at an excitation wavelength of 488 nm and 561 nm, and the emission signals were detected using an Airyscan bandpass filter at 495–620 nm. Hoechst 33342 was illuminated at an excitation wavelength of 405 nm and detected using an Airyscan bandpass filter at 420–480 nm. Z-stack images were obtained at 0.23 μm intervals using an objective lens (Plan-Apochromat, 40×, 0.95 NA). All measurements of live cells were performed in a Tokai incubator (Japan, Tokai Hit Co.) at 37 °C in an atmosphere containing 5% CO_2_. Fluorescence images were processed and 3D-rendered using Zen 2.3 SP1 software (Carl Zeiss, Jena, Germany). The surface function of the Zen 2.3 SP1 software was also used when required.

### 3.16. Fluorescence Correlation Spectroscopy (FCS)

All FCS measurements were performed at 25 °C on a LSM780 microscope (Carl Zeiss, Jena, Germany), as previously described [45]. Doxorubicin was excited at 488 nm, with power minimized by the adjustment of an acousto-optical tunable filter. For the collection of Doxorubicin emissions, the detection pinhole was fixed at a 34 µm diameter (1 Airy), and the emission spectrum was band-pass filtered to 550 to 600 nm. The FCS setup was calibrated daily against FCS measurements of a 10–7 M rhodamine 6G solution. FCS measurements of each cell sample prepared on a Lab-Tek (Nalge Nunc International; Rochester, NY, USA) chambered coverglass was captured for 20 s for 10 replicates, with an interval of 2 s to detect the absolute fluorescence intensity of doxorubicin in the culture medium.

### 3.17. ATPase Activity Assay

Inorganic phosphate generated by ATP hydrolysis, which is linked to transporters, was determined using a colorimetric ATPase assay kit (Sigma-Aldrich; St. Louis, MO, USA). Briefly, Huh-7 cells were incubated at 37 °C for 24 h with DL-SS-MC, DL-MC, free DOX (equivalent DOX dose, 2 µM), or PBS (control) in 10% FBS RPMI 1640 medium. The assay was conducted in accordance with the manufacturer’s instructions. Briefly, samples were incubated for 1 h at room temperature with 30 μL reaction mix reagent, which contained assay buffer and 4 mM ATP. Afterward, 200 μL reagent was added to each well and incubated at room temperature for an additional 30 min to terminate the enzyme reaction and to generate the colorimetric product. The concentration of free phosphate was read at 620 nm. ATPase activity was determined using the following equation: ATPase activity (μM/min) = ([Pi] (μM) × 40 μL)/(5 μL × 60 min). [Pi] = concentration of phosphate in the sample.

### 3.18. Statistical Analysis

Data were analyzed by either one-way or two-way ANOVA and presented as the mean ± standard deviation of three independent experiments. Multiple comparisons among groups were performed using Tukey’s post-hoc test. Statistical analyses were carried out using GraphPad PRISM 9 software (San Diego, CA, USA), and *p* < 0.05 was considered statistically significant.

## 4. Conclusions

In conclusion, we present a strategy for cancer treatment based on vitamicelles with pH and GSH-responsive degradability. Micellar formulations containing different ratios (*w*/*w*) of amphiphilic vitamin conjugates were generated and optimized to ensure the stability of DOX encapsulation. The excellent biocompatibility and rapid biodegradability of VT-SS-MC suggest a great potential as a carrier for anticancer drugs. We show that DL-SS-MC exerts a marked anticancer effect via rapid drug release and efficient inhibition of drug efflux. By exploiting these advantages, carriers can form the basis of a promising strategy to circumvent existing challenges to the delivery of DOX, thereby improving the prospects of DOX-induced chemotherapy. Future studies will examine the efficacy of vitamicelles in xenograft models to confirm that in vitro results are duplicated in vivo.

## Figures and Tables

**Table 1:** Changes in the CMC of vitamicelles as a result of incorporating VE-VB into VE-SS-VB at different weight ratios.

VE-SS-VB:VE-VB Ratio (*w*/*w*)	CMC (μg/mL)	Stability	Vitamicelle
0:1 (VE-VB only)	43.2	Stable	VT-MC
1:1	138.9	Stable	VT-SS-MC
1:2	170.3	Unstable	N/A
1:0 (VE-SS-VB only)	201.1	Unstable	N/A

## Supplementary Materials

All data are available online at https://www.mdpi.com/article/10.3390/ijms222011298/s1.
