# Supplementary material for: Smart Vitamin Micelles as Cancer Nanomedicines for Enhanced Intracellular Delivery of Doxorubicin
Source: Int J Mol Sci. 2021 Oct 19;22(20):11298. doi: 10.3390/ijms222011298 (PMC8538907; doi:10.3390/ijms222011298)
Supplement: Supplementary file 1 [file ijms-22-11298-s001.zip › ijms-1409942-supplementary.pdf]

Supplementary Materials

Smart Vitamin Micelles as Cancer Nanomedicines for Enhanced Intracellular Delivery of Doxorubicin

Na Re Ko [1,2], Sang Ju Lee [2], Arun Pandian Chandrasekaran [3], Apoorvi Tyagi [3], Suresh Ramakrishn [3,4], Seog-Young Kim [5], Do Won Kim [1], Chan-Gi Pack [5] and Seung Jun Oh [2,*]

Determination of the critical micellar concentration (CMC) using an NR probe

A stock solution of vitamin conjugate in THF at 2 mg/mL and a stock solution of NR in THF (5 mg/mL) were prepared. Water (10 mL) was added drop-wise to a series of mixtures comprising the same amount of NR (0.5 mg) and various amounts of vitamin conjugate in THF. The dispersions were stirred at room temperature to evaporate the THF and then filtered to remove excess NR. A series of NR-loaded micelles at concentrations ranging from 5.0 × 10^{-6}–0.6 mg/mL was generated, and their fluorescence spectra were recorded at λ_{ex} = 480 nm and maximum λ_{em} = 640 nm.

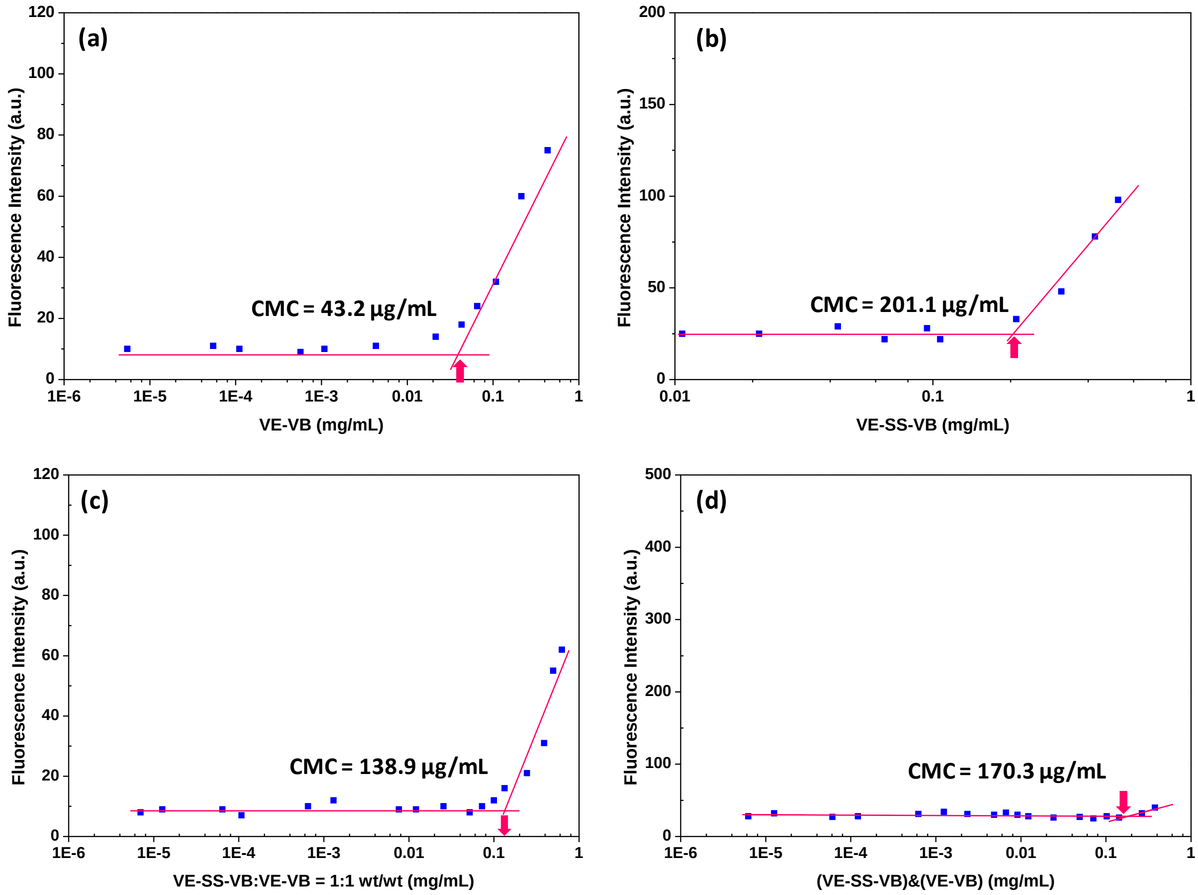

Figure S1. Fluorescence intensity of NR used to determine the CMC of micelles comprising VE-VB only (a), VE-SS-VB only (b), and VE-SS-VB:VE-VB at 1:1 (c) and 1:2 (wt/wt) (d).

Table S1. Long-term stability of the vitamicelles as comparison of particle size measured by DLS. Micelles were stored at room temperature.

Vitamicelles	time = 0	time = 2 months
VT-SS-MC	265.4 ± 1.8	270.9 ± 3.7
VT-MC	274.9 ± 3.6	249.5 ± 2.3
DL-SS-MC	324.1 ± 3.6	314.0 ± 3.4
DL-MC	454.1 ± 6.2	432.5 ± 1.7

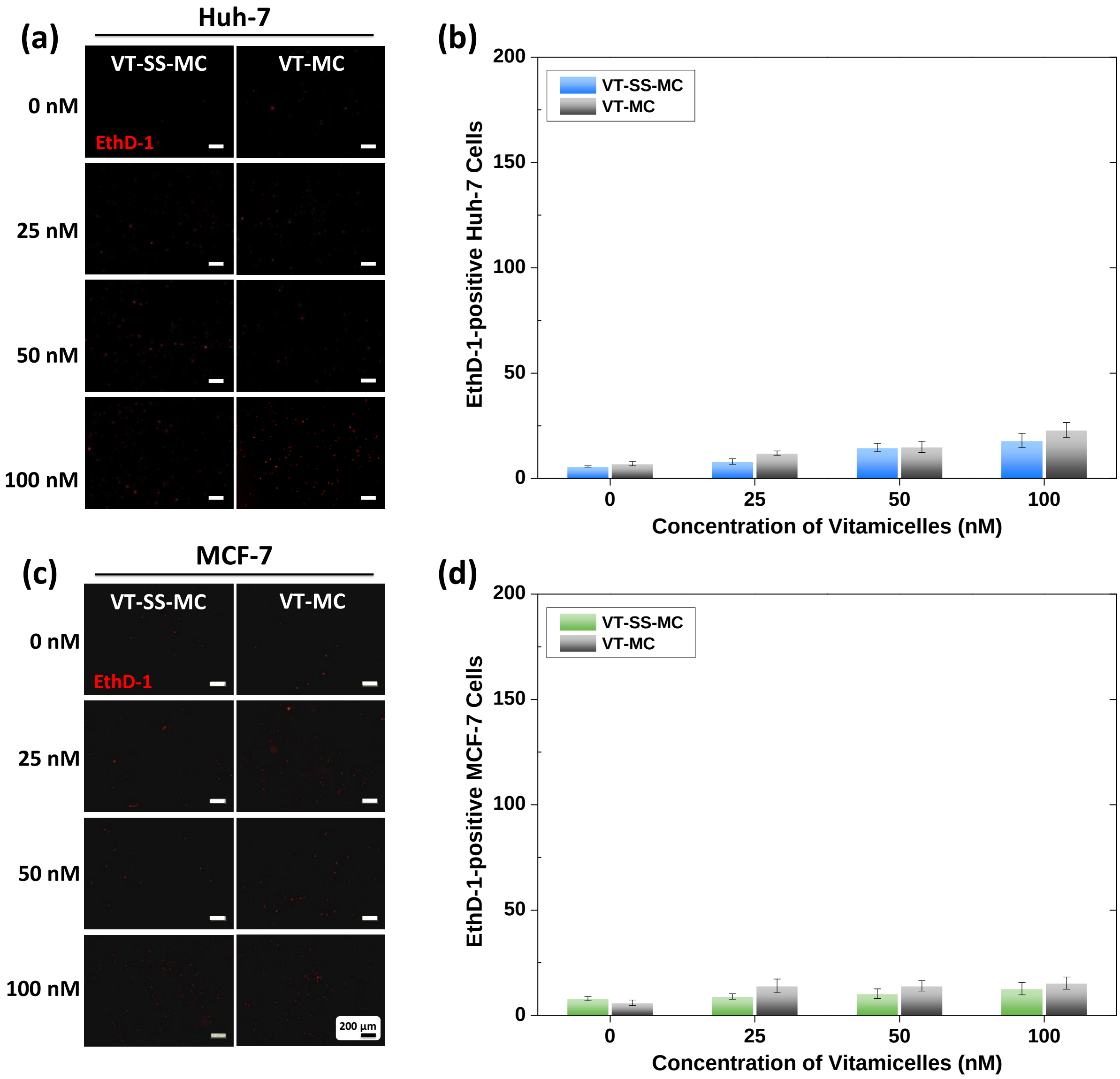

Figure S2. Fluorescence microscopy images of EthD-1-stained Huh-7 cells (a) and MCF-7 cells (b) incubated with empty vitamicelles (VT-SS-MC or VT-MC) for 24 h (scale bar = 200 µm). The dead Huh-7 (b) and MCF-7 (d) cell populations were quantified.

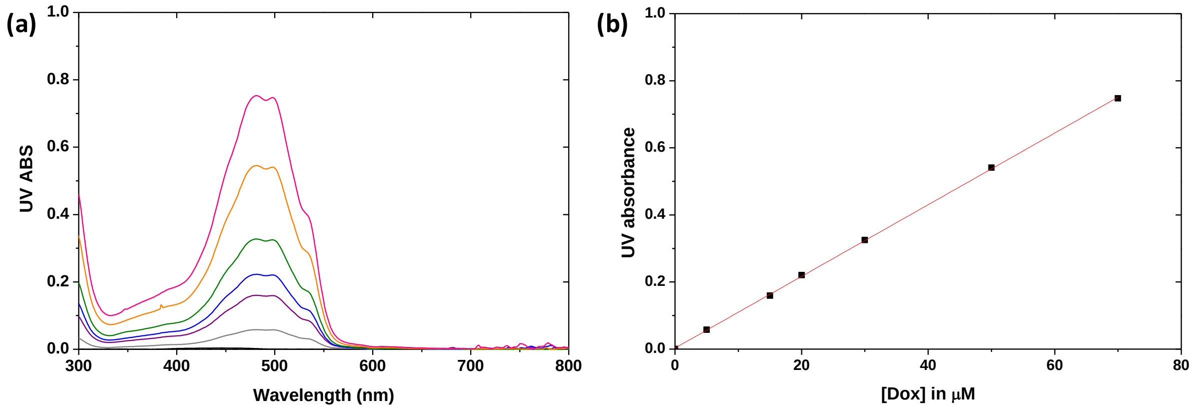

Figure S3. Overlaid UV spectra (a) and absorbance (at λ_{max} = 498 nm) (b) of DOX at various concentrations (μM) in a mixture of water/DMF (1/3 v/v) (used to construct a calibration curve).

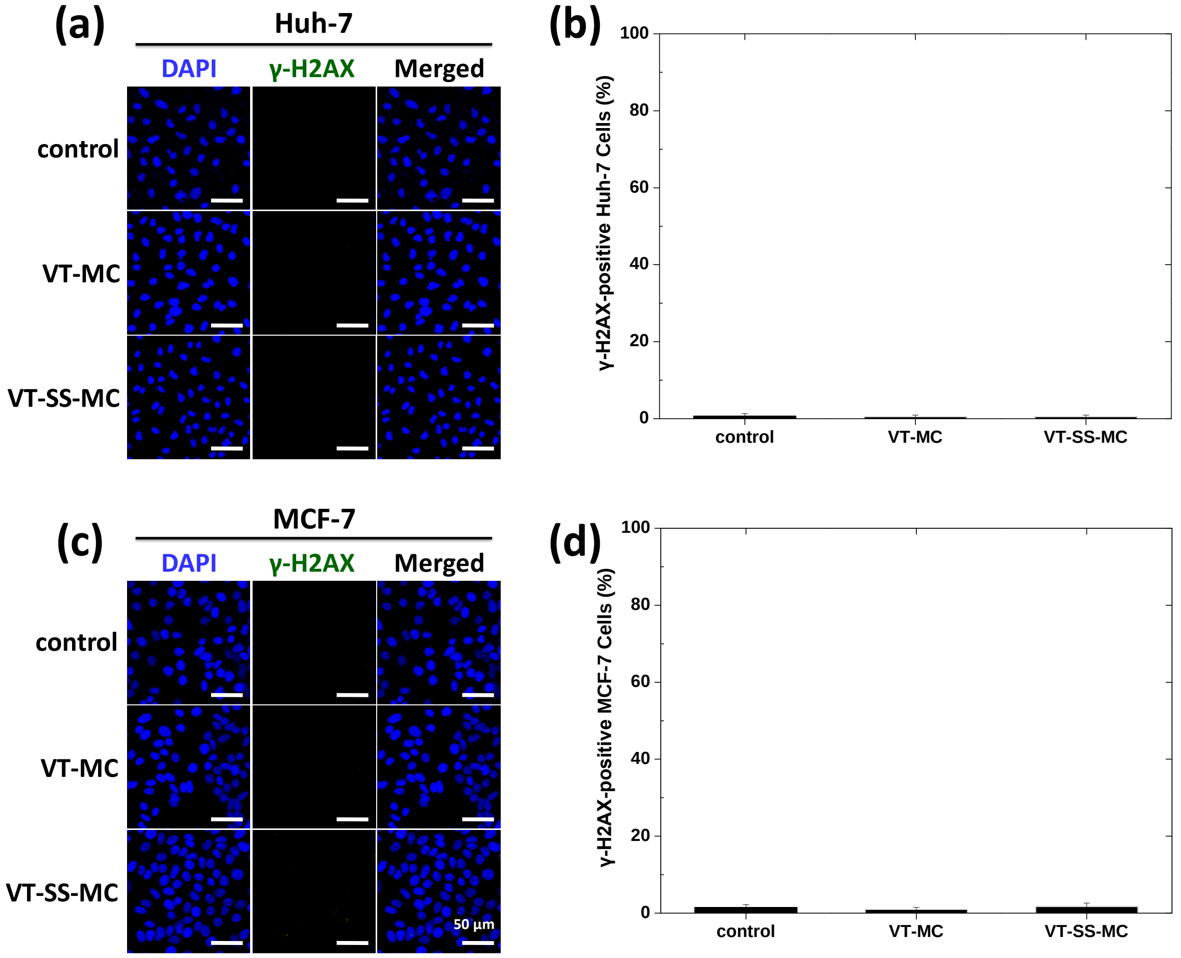

Figure S4. Fluorescence microscopy images of Huh-7 (a) and MCF-7 cells (c) incubated with VT-SS-MC and VT-MC for 24 h. Blue and green fluorescence represents cell nuclei (DAPI) and a marker of DNA double-strand breaks (γ-H2AX). γ-H2AX-positive Huh-7 (b) and -MCF-7 cells (d) were quantified.
